# Anti-Inflammatory Diets and Fatigue

**DOI:** 10.3390/nu11102315

**Published:** 2019-09-30

**Authors:** Ulrike Haß, Catrin Herpich, Kristina Norman

**Affiliations:** 1 Department of Nutrition and Gerontology, German Institute of Human Nutrition Potsdam-Rehbrücke, Arthur-Scheunert-Allee 114-116, 14558 Nuthetal, Germany; cherpich@uni-potsdam.de; 2 Institute of Nutritional Science, University of Potsdam, Arthur-Scheunert-Allee 114-116, 14558 Nuthetal, Germany; 3 Research Group on Geriatrics, Charité–Universitätsmedizin Berlin, Corporate Member of Freie Universität Berlin, Humboldt-Universität zu Berlin and Berlin Institute of Health, 13347 Berlin, Germany

**Keywords:** chronic fatigue, myalgic encephalomyelitis, inflammation, cytokines, anti-inflammatory nutrition, omega-3 fatty acids, polyphenols, probiotics, fatigue reduction diet, cancer

## Abstract

Accumulating data indicates a link between a pro-inflammatory status and occurrence of chronic disease-related fatigue. The questions are whether the observed inflammatory profile can be (a) improved by anti-inflammatory diets, and (b) if this improvement can in turn be translated into a significant fatigue reduction. The aim of this narrative review was to investigate the effect of anti-inflammatory nutrients, foods, and diets on inflammatory markers and fatigue in various patient populations. Next to observational and epidemiological studies, a total of 21 human trials have been evaluated in this work. Current available research is indicative, rather than evident, regarding the effectiveness of individuals’ use of single nutrients with anti-inflammatory and fatigue-reducing effects. In contrast, clinical studies demonstrate that a balanced diet with whole grains high in fibers, polyphenol-rich vegetables, and omega-3 fatty acid-rich foods might be able to improve disease-related fatigue symptoms. Nonetheless, further research is needed to clarify conflicting results in the literature and substantiate the promising results from human trials on fatigue.

## 1. Introduction

Fatigue is described as an unusual overwhelming tiredness which is not comparable to physiologic exhaustion after physical or mental effort and which cannot be recovered by restful sleep [1]. It can show temporary or chronic (>6 months) dimensions [1]. The etiology is not yet fully understood but is probably of heterogeneous origin [2,3]. Fatigue can occur within psychiatric, i.e., depressive, disorders [4], as well as in patients with non-neurological diseases such as fibromyalgia/chronic widespread pain, or in diseases accompanied by chronic (low-grade) inflammation, e.g., cancer, inflammatory bowel disease (IBD), and various other auto-immune diseases [1,5]. Among these patient populations, disease-related fatigue can be experienced by up to 90% of the individuals, with patients with cancer reporting the highest prevalence during and after treatment [1,6]. 

Although disease-related fatigue usually occurs in the diseased population, the percentage of almost healthy community-dwelling adults experiencing considerable pathological fatigue is estimated to be nearly 40% [1], and is more often present in women than men [7,8]. This unexplained, idiopathic, and generalized or chronic fatigue syndrome (CFS), also known as myalgic encephalomyelitis (ME), typically emerges spontaneously with flu-like symptoms [3]. It is characterized by a cluster of unspecific clinical symptoms such as cognitive dysfunction (“brain fog”), headaches, enlarged and painful lymph nodes, irritable bowels, unrefreshing sleep, sore throat, muscle/joint pain or morning stiffness, and severe unphysiologic postexertional malaise [3,9], which simultaneously serve as diagnostic criteria published by Centers of Disease Control and Prevention [10]. Furthermore, if fatigue becomes chronic, disturbed innervations and loss of motor neurons can cause impaired muscle function followed by severe functional limitations [1,4]. Atrophic muscle might thus augment fatigability next to neurologic or peripheral origins, resulting in a vicious cycle. This makes muscle force assessment a relevant diagnostic criterion [1,11]. Perceived fatigue severity can, for example, be assessed with the Chalder Fatigue Scale as a reliable and valid 11-item questionnaire [12]. However, multiple fatigue scales are available [13]. 

As the prevalence of chronic fatigue is high and has considerable impact on the patient’s livelihood and social wellbeing, an effective but well tolerated therapy is urgently required. Treatment options comprise pharmaceuticals, psychological therapy, and exercise programs [14]. However, these are limited in efficacy, could even have negative side effects, and may not be suitable for all types of fatigue. Therefore, this narrative review focuses on promising dietary strategies which are supposed to improve fatigue severity.

## 2. Methods

A comprehensive literature search in PubMed was conducted from inception to August 2019 in order to identify clinical studies which examined anti-inflammatory nutrients, foods, or diets on chronic fatigue. The following key words were used either alone or in combination: fatigue, myalgic encephalomyelitis, omega-3, omega-6, eicosapentaenoic acid, docosahexaenoic acid, arachidonic acid, linoleic acid, eicosadienoic acid, anti-oxida*, vitamin, polyphenol, isoflavone, amino acid, protein, whey, glutamine, probiotic, prebiotic, dietary fibre, ginseng, ginger, zingiber officinale, Nordic diet, Mediterranean diet, Scandinavian diet, Baltic sea diet, anti-inflammat*, inflammatory, and inflammation. Initially, this yielded more than 800 results. Only articles in the English language were chosen. Relevant studies identified from references were added manually. In the end, a total of 21 human trials were critically evaluated.

## 3. Anti-Inflammatory Dietary Strategies 

In healthy conditions, inflammation is a normal, self-limiting, and well controlled response to infections, and is thus not damaging, but helpful to protect the host. However, since fatigue is frequently associated with conditions characterized by immune deficiency and/or over-activation of inflammation [15,16], the chronic pro-inflammatory status of these patients is believed to cause or aggravate the symptoms of fatigue [17,18]. In IBD, for example, microbial dysbiosis is suspected to promote gut leakage and bacterial translocation which, in turn, induces an abnormal immune response and subsequently initiates a pro-inflammatory status [19,20]. Dysfunction of T-/B-cell memory and natural killer cells [21,22], chronic activation of innate antiviral signaling pathways (e.g., protein kinase R), different anti-oxidative (e.g., glutathione peroxidase, catalase) as well as increased inflammatory markers (e.g., C-reactive protein (CRP), tumor necrosis factor alpha (TNF-α)) [17,23], and increased activation of nuclear factor kappa beta (NF-κB) [24,25] are frequently described pathways in fatigue [1,26,27]. Interleukin (IL)-6, in particular, has been linked to fatigue and is even called the “sleep factor”, which could thus partly explain the characteristic tiredness [28] (p. 207). 

**Figure 1:**
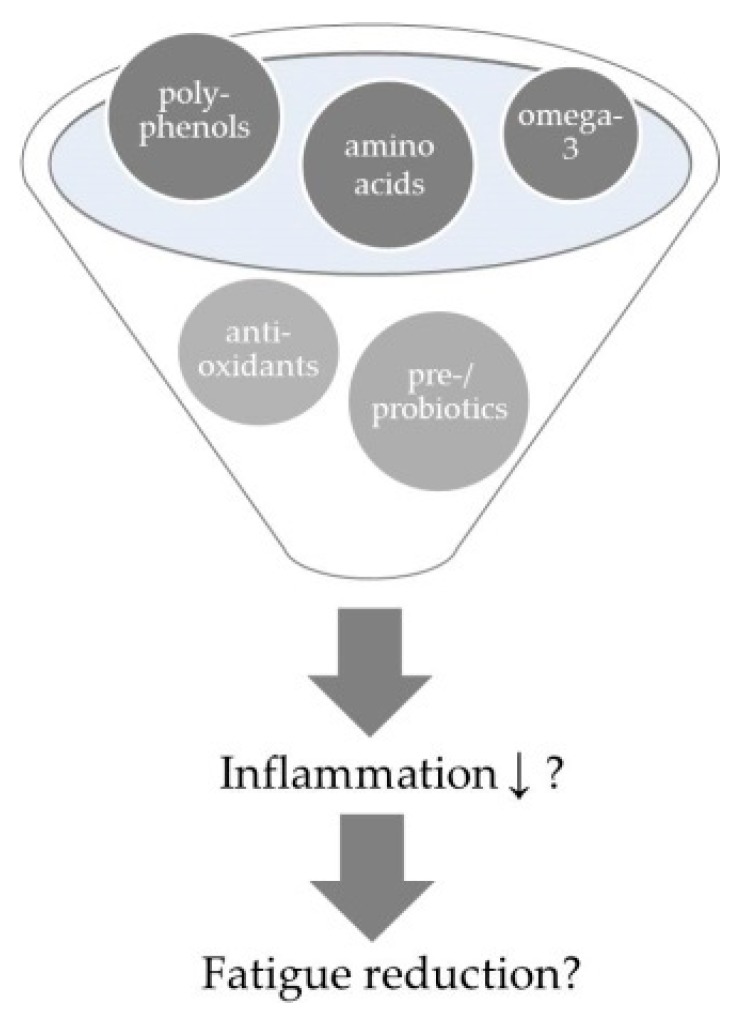
Accumulating data indicates a link between a pro-inflammatory status and occurrence of chronic disease-related fatigue. The leading questions are whether the observed inflammatory profile can be reduced (↓) by anti-inflammatory nutrients or diets, respectively and if this improvement in turn is translated into a significant fatigue reduction.

It appears that the occurrence of chronic disease-related fatigue is related to a pro-inflammatory status. The leading question is not only whether the inflammatory profile can be improved by nutrients with suggested anti-inflammatory properties, but also whether fatigue symptoms can in turn be affected (Figure 1). Different foods and nutrients which have been suggested to positively affect these pro-inflammatory pathways/markers are described in the following sections. Consequently, anti-inflammatory diets, which contain and combine several of those anti-inflammatory nutrients, are believed to work even better than single nutrients or foods. They will be reviewed in further sections. Table A1 gives a summary of evaluated human trials in this field.

### 3.1. Anti-Inflammatory Single Nutrients 

#### 3.1.1. Poly-Unsaturated Fatty Acids 

The inflammatory status offers a pathophysiological background for therapeutic use of omega-3 fatty acids (FA) [29], which belong to the family of polyunsaturated fatty acids (PUFA) and are known for their anti-inflammatory properties. Detailed pathways have been reviewed extensively elsewhere [30,31]. The formation of either anti-inflammatory omega-3 FAs, like eicosapentaenoic acid (EPA) and docosahexaenoic acid (DHA), or omega-6 FAs with rather pro-inflammatory properties [31], is competitive through shared desaturase pathways and depends on substrate availability which is mainly determined by dietary habits. An insufficient incorporation of phospholipids in the membranes as a key aspect [30] is not only expected to result in inadequate neurotransmissions and decreased brain function, but also impaired immune-inflammatory responses [32,33]. 

The expected anti-inflammatory and assumed fatigue-improving effects of omega-3 FAs have been examined in different studies. Metabolomics within the Twins UK cohort showed that reduced circulating EPA levels are significantly associated with fatigue in women with fibromyalgia [34]. A low omega-3 index (i.e., low incorporation of omega-3 FAs in erythrocytes in relation to omega-6 FAs) has been measured in a small Spanish patient population with CFS [35]. Furthermore, compared to healthy controls, a significantly diminished omega-3:omega-6 ratio but higher levels of oleic acid, linoleic acid (LA), and arachidonic acid (AA) can also be detected in another sample of patients with CFS [36]. In this patient population studied by Maes et al., omega-3:omega-6 ratios (<0.15) had good diagnostic value with a sensitivity/specificity of 72.7%/91.7% and were also negatively correlated with fatigue severity (r = −0.46, *p* = 0.027). Since zinc is important for desaturase activity and thus biosynthesis of these long-chain PUFAs [37], the positive correlations found between omega-3:omega-6 ratios and serum zinc levels in CFS patients are plausible (r = 0.56, *p* = 0.009) [36]. 

Observational analyses in breast cancer survivors indicate inverse associations between low omega-3 FA and high omega-6 FA intakes, higher inflammation levels, and worse fatigue scores [38]. A randomized controlled trial (RCT) tested the effects of omega-3-enriched oral nutritional supplements (ONS) with 2.2 mg EPA daily [39] in a group of 84 patients with non-small cell lung cancer. After two cycles of chemotherapy, significantly lower fatigue (*p* = 0.04) was observed in the intervention group. However, inflammatory markers, such as IL-6 and TNF-α, did not change significantly between groups, and CRP showed only borderline significant improvements (*p* = 0.07). Another clinical trial in 332 cachectic patients with cancer could not confirm that supplementation of EPA rich ONS (2.2 g/day) was able to significantly improve fatigue symptoms or inflammatory markers when given as a sole therapy within a 5-arm study design [40]. Only combined therapy (daily EPA-enriched ONS plus 320 mg megestrol acetate or 500 mg medroxiprogesterone acetate, 4 g L-carnitine, and 200 mg thalidomide) lead to significant improvements in fatigue symptoms (*p* = 0.047) and IL-6 (*p* = 0.0187) in this cohort after 4 months of intervention [40]. Though a Cochrane review investigated 27 systematic reviews concerning management of fatigue and unintentional weight loss in patients with advanced illness, the authors identified only one review which surveyed the efficacy of EPA in cachectic patients with cancer [41], which also did not find convincing evidence for fatigue reduction [42]. 

A multicenter RCT compared 6-week supplementation of high-dose omega-3, omega-6, and low-dose combination of omega-3:omega-6 in 97 breast cancer survivors on fatigue [43]. High-dose omega-6 supplementation reduced fatigue symptoms more than high-dose omega-3 (effect size = −0.86, *p* < 0.01) and combined omega-3:omega-6 supplementation (effect size = −0.20, *p* = 0.048). Furthermore, significant ameliorations of inflammatory load, especially reductions in TNF-α and CRP, with high-dose omega-6 compared to omega-3 or combined were observed. As these results are in contrast to the original hypothesis that omega-6 FAs are rather pro- instead of anti-inflammatory, the authors proposed that the observed improvements in disease-related fatigue are possibly stronger connected to TNF-α and CRP, and less linked with other inflammatory markers as, for example, interferon gamma, which are recognized as major mediators of cancer-specific inflammation [43]. Unfortunately, these markers were not reported by the authors [43]. 

Rheumatoid arthritis (RA) is an auto-immune disease that typically affects the lining of the joints. Fatigue is a major complaint and experienced by up to 90% of patients with RA in exacerbation [13]. In 2013, Cramp et al. published a Cochrane review on 24 studies evaluating the benefits of non-pharmacological treatments for fatigue in RA [44], which included one trial on the effects of omega-3 FA supplementation in combination with indomethacin [45]. As fatigue was referred to as “vitality” on the SF-36 (a widely used quality of life (QoL)-measurement) and significant differences between groups were not reported here [45], the quality of this trial was deemed rather low, and the conclusion was that there is insufficient evidence for omega-3 FA supplementation in patients with RA [44]. 

Systemic Lupus Erythematosus (SLE) is another auto-immune disease accompanied by inflammation which can affect nearly all body tissues [46]. Fatigue is highly frequent in this patient group [46] and is again linked to higher inflammatory and lower omega-3 status [47,48]. Arriens et al. evaluated the impact on self-reported QoL, disease activity, and some inflammatory markers (erythrocyte sedimentation rate (ESR), IL-12, IL-13) after 6 months of placebo-controlled omega-3 fish oil intervention (2.25 g EPA and 2.25 g DHA vs. refined olive oil) in 32 patients with active SLE [49]. While a significantly improved inflammatory profile was shown in the fish-oil group (ESR (*p* = 0.008); IL-13 (*p* = 0.033); IL-12 (*p* = 0.058)), fatigue levels (SF-36 subdimension energy/fatigue (*p* = 0.092) Fatigue Severity Scale (*p* = 0.350)) were not similarly changed. 

Multiple sclerosis (MS) is an auto-inflammatory disease characterized by demyelinated nerve cells in the central nervous system (CNS) and spinal cord. One RCT compared the effects of a 12-month long low fat diet (15 energy%) supplemented with fish-oil (1.98 g/day EPA and 1.32 g/day DHA) against a normal fat diet (≤ 30 energy%) supplemented with olive oil in these patients [50]. A significant reduction in fatigue was seen after 6 months in the fish-oil group (*p* = 0.0348). However, these effects diminished after 12 months in the intervention group and were in fact slightly improved in the control group, though only with borderline significance (*p* = 0.059). Also, a daily oral administration of 2.2 g omega-3 FAs (1.35 g EPA and 0.85 g DHA) alone or in combination with interferon-ß for 6 months has not been effective regarding fatigue (*p* = 0.97) or QoL (*p* = 0.53) in another trial [51]. The omega-3:omega-6-ratio has been checked, but only with regard to relapse rate, which revealed no treatment effects. 

#### 3.1.2. Anti-Oxidative Vitamins 

In vitro analytics displayed that patients with CFS show impaired anti-oxidative capacity and are more prone to peroxidation of (very) low-density lipoproteins, uncovered by lower transferrin levels as well as shortened lag time to retard the initiation of peroxidation [52]. Many patients with CFS use nutritional supplements like vitamins and/or minerals to improve their clinical symptoms [53]. Vitamin A (retinoic acid), for example, is known for its inflammation-balancing effects by suppressing pro-inflammatory T-helper cells as well as the gene expression of different inflammatory cytokines and their transcription factors [54]. This inflammation modulating effect, and its impact on fatigue, has been verified in a double-blind placebo-controlled trial of 101 patients with MS. According to these results, high-dose vitamin A (25,000 international units (IU)/day retinyl palmitate for 6 months followed by 10,000 IU/day for 6 months) significantly reduced fatigue scores (*p* = 0.004) after 1 year of intervention [55]. However, a recent meta-analysis including 27 trials explored vitamin and mineral status in patients with CFS in comparison to healthy controls as well as supplementation in physiological amounts and concluded that robust evidence is lacking to support vitamin supplementation [53]. Moreover, the authors doubt that vitamin and mineral deficiency plays a significant role in the pathophysiology of CFS. Nonetheless, these results might reflect differences between the etiology of disease-related fatigue and fatigue within an idiopathic syndrome. 

#### 3.1.3. Vitamin D (Cholecalciferol)

One essential mechanism in inflammatory pathways is the sustained activation of NF-κB. This upregulated NF-κB activity and related stimulation of pro-inflammatory mediators has already been extensively examined in patients with CFS [24,25]. Vitamin D, in its active form known as 1,25-dihydroxyvitamin D3 (calcitriol), is able to directly and indirectly counterbalance the activation of NF-κB and its products [56]. Furthermore, it is able to balance the redox system, to regulate immune tolerance, and to support the innate immune response [56,57]. The anti-inflammatory properties and immune-modulating effects of vitamin D are also regulated by its enhancing influences on skin and the mucosal barrier, which lowers the risk of microbial invasion [57]. 

Results from epidemiologic studies in different patient samples are not consistent. Some indicate low vitamin D levels in fatigue [58,59] while others do not [60,61]. Nonetheless, Johnson et al. described in a case report how they were able to improve unexplained fatigue in a 61-year-old man with high-dose vitamin D (50,000 IU/week for 8 weeks followed by 1000 IU/day) [62]. Serum levels increased from 18.4 ng/ml to 27.2 ng/ml after 3 months and to 32.2 ng/ml after 12 months. Fatigue dissolved completely within 3 months parallel to increasing serum vitamin D levels. A systematic review by Sousa et al. elucidated the effect of vitamin D supplementation in SLE in four RCTs [63], but only one RCT evaluated the effects of vitamin D supplementation on fatigue, which revealed positive effects in young adults with juvenile SLE [64]. Supplementation of 50,000 IU/week for 24 weeks not only increased serum levels (*p < 0*.001) and decreased disease activity (*p* = 0.011), but did also decrease fatigue global score (*p* = 0.012) with particular advancement in social life aspects (*p* = 0.008). 

#### 3.1.4. Polyphenols 

Polyphenols are aromatic secondary metabolites which are able to protect plants against pathogens. Over 8,000 polyphenols have already been identified which can be divided into different classes according their chemical properties and functions (e.g., radical scavenger), of which phenolic acids, tannins, and flavonoids are the most abundant ones found in foods [65]. 

Isoflavones, belonging to flavonoids and mainly found in soy and soy products, are highly bioavailable and are described to have anti-oxidative and anti-inflammatory functions [66]. They are assumed to play a useful role in the management of CFS [67]. At least in animal studies, isoflavone treatment with daidzein and genistein reversed the polyinosinic:polycytidylic acid-induced reduction in locomotor activity (as an indicator for fatigue) and the increment of pro-inflammatory status [67]. However, human studies examining the effects of isoflavones on chronic fatigue are missing. 

The polyphenol epigallocatechin gallate (EGCG) is known as the active component of green tea and recognized to have anti-inflammatory properties next to anti-carcinogenic and anti-oxidant activity [68]. Studies on mice with experimental CFS models have indicated that EGCG is potent in reducing lipopolysaccharide (LPS)-induced inflammatory and oxidative levels, as well as ameliorating fatigue measures [68,69]. Again, no human studies investigating the effect of EGCG on disease-related fatigue have been identified in this literature search. 

The flavonol quercetin, a yellow natural dyestuff in many fruits and herbs, has been investigated for its anti-inflammatory effects in animal models and different cell lines [70], as well as in humans [71]. The beneficial effect is mainly based on its adenosine monophosphate-activated protein kinase stimulating activity [72] which inhibits NF-κB and inflammatory pathways [73]. Animal models are contradictory in their results regarding the role of quercetin in preventing cancer-related pro-inflammatory levels and fatigue [74,75], and large-scale human trials are not yet available. One case study reported partial improvement in fatigue after daily supplementation of 1,000–2,000 mg quercetin in combination with 100 mg cyclophosphamide in a patient treated for metastatic bladder cancer [76]. However, the effects did not last for the whole treatment period and it has to be considered that the interim observed improvements in fatigue might have resulted from the complete response to cancer treatment. On the other hand, an uncontrolled phase I trial revealed not only a safe daily supplementation of 450 mg or 900 mg isoquercetin for a median of 81 days (range 75.5–86.5) in 12 patients with kidney cancer receiving 50 mg sunitinib/day, but also a significant reduction in fatigue scores (*p* = 0.002) [72]. However, inflammatory profiles were not examined and considering the uncontrolled study design, the results have yet to be verified in larger scale RCTs. To sum up, regardless of their known anti-inflammatory effects, none of these promising polyphenols have been convincingly proven to likewise reduce fatigue symptoms in humans until now, and dose-dependent relationships have to be taken into account. Moreover, in view of low concentrations in normal diets, probably relatively high doses have to be supplemented to gain therapeutic value [77].

#### 3.1.5. Protein/Amino Acids

A low protein intake is indicative of a higher risk of cancer-related fatigue (CRF) [78]. Maintaining muscle mass and strength through higher, or at least adequate, protein consumption may enhance fatigue by preserving functional capacities. Especially, whey protein, which is rapidly digestible and high in leucine, is not only effective in stimulating muscle protein synthesis in the young and old [79,80], but is also hypothesized to have anti-oxidative and anti-inflammatory properties [81]. Furthermore, plant-based proteins like soy have anti-inflammatory effects, though this could be also traced back to the high isoflavone content [66,67].

A lack of amino acids has been implicated in the development of disease-related fatigue. For example, in neuro-inflammatory conditions, chronic tryptophan deficiency (as an indicator for protein malnutrition) has been suggested to be relevant in the development of CFS [82], since the kynurenine pathway and subsequent serotonin and energy production is altered. Ringseis et al. delineated that carnitine is positively affecting inflammatory processes, even under pathological conditions like cancer [83]. Though an RCT in patients with CFS within a 3-arm design revealed significantly affected mental fatigue (*p* = 0.015) after daily supplementation of 2 g acetyl-L-carnitine alone for 24 weeks, the treatment did not affect general (*p* = 0.218) or physical fatigue scores (*p* = 0.313) [84]. Indeed, within the other treatment arms, either acetyl-L-carnitine in combination with propionyl-L-carnitine (*p < 0*.0001) or 2 g propionyl-L-carnitine alone (*p* = 0.004) improved general fatigue in a significant manner [84]. As different pathologies between disease-related and idiopathic fatigue again can be assumed, this beneficial treatment could not be verified in a randomized phase III clinical trial in patients with CRF, which revealed that oral L-carnitine therapy alone for 4 months was neither effective in reduction of inflammatory markers nor perceived fatigue [40]. Furthermore, a meta-analysis including 12 trials concluded that fatigue cannot be treated effectively with L-carnitine supplementation in patients with cancer [85]. Moreover, adverse events have to be taken into account when administering high doses of L-carnitine [86].

Following metabolic profiling, glutamine has been identified as significantly depleted in patients with CFS compared to healthy controls [87]. The body pool of amino acids, mainly stored in the muscles, in large part consists of glutamine. This amino acid is very multifaceted as it can be used for gluconeogenesis and de novo synthesis of amino acids and proteins amongst other metabolically relevant functions. Further, glutamine is called the “nitrogen shuttle”, as it transports one third of all nitrogen in the blood derived from amino acids [88] (p. 189). In immune-related catabolic conditions, muscles show a high glutamine output to feed lymphocytes and macrophages. This makes this semi-essential amino acid become essential [88]. Furthermore, glutamine acts on the production of pro-inflammatory cytokines of human mucosal cells [89]. This could possibly explain how glutamine supplementation can positively affect mucositis (inflamed mucosal layer) in patients with cancer during treatment [90] and how this may contribute to CRF. A cross-sectional investigation observed that deprived glutamine levels are not only associated with a poor nutritional status (*p* = 0.0438), but also fatigue severity (*p* = 0.0303) and inflammation (CRP; *p < 0*.0001) in patients undergoing cancer treatment [91]. Unfortunately, controlled clinical trials in humans investigating these potential effects of glutamine on disease-related fatigue are lacking. 

### 3.2. Anti-Inflammatory Foods

#### 3.2.1. Probiotics

Patients with IBD suffer more often from CFS than healthy controls [19,92] and patients with CFS often suffer from gastrointestinal disturbances [93,94]. Pathogens or microbes that cause dysbiosis can alter the human immune system and dysregulate mitochondrial functioning in a way that complicates host response and inflammatory signaling, which could contribute to (persistence of) CFS (for detailed pathways, see [95]). In this context, it is also proposed that the gut-brain-axis is strongly involved in CFS, including microbial communication that could affect CNS activity and, through this, promote sickness behavior [95]. An overload of neurotoxic ammonia- and D-lactic acid-producing gut bacteria could possibly further explain CNS manifestations in CFS. Interestingly, these bacteria have been shown to be over-represented in patients with CFS [96]. Additionally, Giloteaux et al. detected different bacterial composition in the gut between healthy subjects and patients with CFS, which is even comparable to the intestinal flora of patients with IBD [20]. This different composition has been translated into lower and less diverse colonization of microbiota which support an anti-inflammatory status (firmicutes phylum like bifidobacteria), and at the same time, higher proliferation of rather pro-inflammatory microbiota which also promote gut leakage (e.g., proteobacteria) [20]. These observations have further been confirmed through blood analysis. Patients with CFS showed significantly higher blood levels, indicative of bacterial translocation (higher IgM and IgA levels following LPS, LPS-binding protein (LBP), LPS/LBP receptor sCD14) [20,97]. The LPS-induced NF-κB activity might, in this case, be both the cause and consequence of a weakened tight junction barrier [98].

Lactic acid bacteria have been proposed as a treatment for disturbed microbiota in patients with CFS [99]. Animal models revealed that they are potent in preventing immune-modulating and inflammatory processes, for example by increasing dendritic and regulatory T cells [100] as well as decreasing TNF-α levels [101]. Furthermore, diets high in dietary fibers are associated with reduced pro-inflammatory profiles [102] and are moreover inversely correlated with fatigue occurrence (r = −0.38, *p* < 0.05) [103]. Their positive effects can be linked to an improved composition of protective gut microbiota [104]. However, human interventional trials with convincing evidence are scarce. A systematic review revealed only two human trials with probiotics (*Lactobaciulls casei* and *Bifidobacterium infantis*) in CFS [94]. Although pro-inflammatory levels decreased in the probiotic intervention groups, no further investigations into the effects on fatigue have been made. One recently published placebo RCT demonstrated a dose-dependent amelioration of perceived fatigue (*p* = 0.037) after 8 weeks of *Lactobacillus* and *Bifidobacterium* supplementation in patients with irritable bowel syndrome (IBS) [105]. Other trials with supplementation of various probiotic mixtures in patients with CFS for 10 weeks [106], colorectal cancer [107] and primary sclerosing cholangitis with IBD for 12 weeks [108], or spondyloarthritis for 4 weeks [109] were not successful in significantly improving the fatigue status. Finally, a systematic review, including 25 studies in patients with CFS and IBS, concluded that there is yet insufficient “evidence available for the therapeutic use of probiotics in the CFS/ME patient group, although some limited data show potential for alleviating specific subtypes of IBS symptoms in CFS/ME patients” [110] (p. 473). 

#### 3.2.2. Root Plants 

The consumption of Panax ginseng is not restricted to Asian countries anymore. This root incorporates ginsenosides and ginseng polysaccharide as main bioactive compounds, which have been proven to affect inflammatory activity and microbial composition [111]. In vitro models, for example, have illustrated the positive effects of ginsgeng polysaccharide by enhancement of microbial deglycosylation and mucosal absorption [112]. Furthermore, fatigue models in mice showed remarkable reductions of chemotherapy-induced inflammatory cytokines which are associated with CRF (TNF-α, IL-6) and simultaneously an increase in fatigue-related markers (forced swimming, wheel running) with purified ginseng extract [113]. However, these positive results from animal and in vitro research could not be transferred to humans. One trial examined the anti-inflammatory and anti-fatigue effects of ginseng in 80 patients with nonalcoholic fatty liver disease [114]. After three weeks, consumption of concentrated ginsenoside capsules resulted in significant decrease of TNF-α (*p* = 0.031) and a fatigue reduction (*p* < 0.001) in the ginseng group, though the observed fatigue reduction was not significantly different to the placebo group (*p* = 0.221). Interestingly, patients with a body mass index ≥25 kg/m^2^ benefited more from the intervention, both at an inflammatory (*p* = 0.010) and a fatigue level (*p* = 0.003). Another human trial of 90 people with idiopathic fatigue and four weeks of either high- or low-dose supplementation of ginseng extract could not show significant changes in global fatigue compared to placebo group [115]. Only subscores for mental fatigue (high-dose (2 g/day): *p* = 0.002; low-dose (1 g/day): *p* = 0.033) and fatigue on a visual analogue scale had an improved effect compared to the placebo (*p* = 0.037).

Ginger (Zingiber officinale) is of widespread use in Asian as well as Western countries. Its active constituents, gingerol and shogaol, have extensively been studied and attributed with a variety of properties, including anti-oxidant and anti-inflammatory properties [116]. One double-blind RCT showed that 1.2 g of ginger extract significantly reduced CRF (*p* = 0.006) and improved QoL (*p* = 0.015) in patients with moderate-to-high risk of chemotherapy-induced nausea [117]. In spite of these positive results which are indicating promising usefulness in fatigued patients, further human trials with ginger investigating fatigue levels are lacking. 

### 3.3. Anti-Inflammatory Dietary Patterns

#### 3.3.1. Fatigue Reduction Diet

A better diet quality is indicative of lower fatigue occurrence and severity in breast cancer survivors [103,118]. Within a cross-sectional investigation, Zick et al. analyzed habitual diets of 40 former breast cancer patients and revealed that consumption of fish, whole grains, and vegetables (particularly leafy greens and tomatoes) was over-represented in the diets of non- or less-fatigued survivors compared to the fatigued ones [119]. Looking further, non-fatigued survivors also had higher intakes of anti-inflammatory (carotenoids, omega-3 FAs) and anti-oxidative (vitamin A and C) nutrients [119]. These observations led to the concept of the so called “fatigue reduction diet”, which is rich in whole grains, vegetables, fruits, and omega-3 FAs. Its assumed positive effects on perceived fatigue have subsequently been verified by the same research group in a pilot study with 30 breast cancer survivors [120]. Following the diet for 3 months resulted in a significantly greater fatigue reduction compared to the control group with standard care (44 ± 39% vs. 8 ± 34%; *p* = 0.01). In accordance with a higher consumption of fibers (whole grains, vegetables, and fruits) and better fat quality, serum levels of carotenoids (cryptoxanthin, zeaxanthin, lutein, and lycopene; all *p* < 0.05), as well as omega-3 FAs (*p* < 0.01) and the omega-3:omega-6 ratio (*p* = 0.02), were significantly increased, while saturated FAs had decreased (*p* = 0.04) [120].

#### 3.3.2. Leaky Gut Diet

Since gut leakage or “intestinal mucosal dysfunction” is recognized as a possible driver of pro-inflammatory pathways and associated chronic fatigue symptoms, the so called leaky gut diet has been composed by Maes et al. to attenuate gut barrier function and, at the same time, reduce LPS-induced immune-inflammatory response [98] (p. 102). This low-carbohydrate diet is free of milk allergens and gluten, and is combined with specific natural anti-inflammatory and anti-oxidative substances (NAIOSs) like glutamine, N-acetyl-L-cysteine, zinc, L-carnitine if needed, coenzyme Q10, taurine, curcumin, or quercetin. Aside from this, the treatment is sometimes complemented with intravenous immunoglobulins [98]. A first case report of a 13-year-old girl with CFS after pharyngitis indicated a continuous improvement of fatigue symptoms until complete remission within two years after first consultation [97]. Maes et al. evaluated clinical outcomes in patients with CFS, who followed an individualized leaky gut diet for 10–14 months in larger non-interventional settings [98,121]. The majority (63.5%) of patients showed a good clinical response, with significant reductions of immunoglobulin (Ig)A and IgM responses to LPS (*p* = 0.0009 and *p* = 0.0002), and significant improvements in fatigue scores (*p* = 0.0002) [98]. The NAIOs appeared to normalize IgA and IgM levels by reducing gut-derived inflammation and prevention of bacterial translocation through enhancing weakened tight junctions [121]. 

#### 3.3.3. Mediterranean Diet 

The Mediterranean diet is characterized by a high consumption of whole grains, legumes, nuts, fish, lean meat, dairy, olive oil, fruits, vegetables, and moderate alcohol consumption, mainly in terms of red wine. This makes the diet a source of high-quality FAs (i.e., omega-3 and omega-9), fibers and complex carbohydrates, vitamins and minerals, as well as secondary plant metabolites [122]. Especially the high amount of phytonutrient-dense, polyphenol-rich foods in this diet is presumed to be a main reason for its advantageous effects, next to fiber content and fat quality [123]. Olive oil, for example, is rich in the omega-9 mono-unsaturated fatty acid oleic acid, which can be converted to eicosatrienoic acid. Eicosatrienoic acid is known as an inhibitor of leukotriene B4 synthesis and might therefore act as an anti-inflammatory FA comparable to fish oil (EPA) [124]. In this context, the Mediterranean diet is believed to improve disease-related fatigue by lowering the inflammatory load and simultaneously balancing gut microbiota [14].

A Cochrane review, which focused on non-pharmacological interventions for fatigue in RA, found one trial that investigated the effects of a Mediterranean diet [44]. Although this trial was able to show benefits in fatigue referred to as “vitality” on the SF-36 [125], the trial quality was deemed rather low, so the authors of the systematic Cochrane review could only conclude that there is no sufficient data for the effectiveness of a Mediterranean diet on fatigue reduction in patients with RA [44]. Although promising due its inflammation-reducing properties, other clinical trials in humans on the effects of a Mediterranean diet on fatigue are lacking.

#### 3.3.4. Nordic Diet

Similar to the Mediterranean diet, the Nordic diet is characterized by high consumption of whole grains and unrefined sugars, fish and lean meat, as well as dairy products, canola or rapeseed oil, and fruits and vegetables known from the northern European countries like berries and cabbage [126]. However, the Nordic diet was conceptualized within a Scandinavian public health project two decades ago to counteract growing obesity [127] and is thus not as established or adopted in Northern regions as the traditional and culturally embedded Mediterranean diet is in Southern regions. In contrast to the Mediterranean diet, more variations in diet composition do also exist between the different Nordic regions. These variances are mainly observed in amount and type of bread, fish, fruits, berries, and vegetables [126]. Nonetheless, due to the comparable beneficial pattern, the Nordic diet is also supposed to have an anti-inflammatory impact by downregulating immune- and inflammation-related markers [128,129]. Although at least in theory conceivable, examinations of fatigue reduction have not been made until now. 

## 4. Discussion

This review summarized currently known anti-inflammatory dietary strategies in various patient populations and their effects on perceived fatigue. Despite the large theoretical rationale from animal and in vitro models for therapeutic use of anti-inflammatory diets in fatigued patients, and also some interesting and promising results from human trials, the evidence in humans is rare. Most of all, systematic reviews, which investigated the effects of different nutritional interventions on idiopathic fatigue as well as disease-related fatigue, share this conclusion [41,53,85,94,110], with the exception of studies in patients with cancer [14,86] or SLE [63]. 

### 4.1. Why are Results of Anti-Inflammatory Dietary Strategies Controversial in Terms of Fatigue Management?

In general, it seems that the longer the chronic fatigue persists, the lower the chances of any treatment benefit are. Conversely, the shorter the duration and the younger the patient, the higher the chances are that fatigue responds to treatment or improves spontaneously [98,130]. Compared to idiopathic fatigue, it appears that disease-related fatigue responds in a different manner to treatment, which is most likely due to the different etiologies [131]. Furthermore, studies and outcomes are difficult to compare, because diverse outcome measures are used [13,46] and fatigability is often described in the context of QoL measurements, which is rather imprecise. Indeed, patient populations, including their pathophysiology, are very heterogeneous, so that fatigue does therefore not always equal fatigue. Diagnosis, as well as classification, of fatigue is still challenging. Moreover, durations of interventions differ, ranging from a few weeks to several months or years. If untested, variability in actual content, quality, and bioavailability of applied supplements could have affected the results, too. On top of this, different material sampling (e.g., serum vs. plasma vs. cerebrospinal fluid), sensitivity of sample analysis (e.g., enzyme-linked immunosorbent assay vs. Multiplex Luminex), and circadian rhythm, as well as day to day variations, have to be kept in mind. Medication use has not always been reported (e.g., non-steroidal anti-inflammatory drugs) which might well confound results. Unreported additional anti-inflammatory supplementation is also possible (e.g., simultaneous intake of omega-3-rich oils or multivitamins), but detailed dietary intakes are seldom available. 

The majority of human trials examining fatigue levels used omega-3-FA supplementation, which appears promising considering the reduction of inflammation. However, only few studies show consistent beneficial influence on fatigue levels. Although fatigue has been suggested to depend on the inflammatory load, only a few interventional trials simultaneously investigated effects on inflammatory markers with fatigue levels. Interestingly, if inflammatory levels were actually measured, they were seldom reduced in the same manner as fatigue levels. Thus, a distinct cause–effect relationship between inflammatory status and fatigue levels yet has to be verified. Besides this, Raison et al. doubt that the activation of the innate inflammatory processes is specific to CFS [132]. Indeed, it seems that CFS is not consequently related to higher pro-inflammatory cytokine release and instead is associated with normal cytokine production comparable to healthy controls [133]. Since CFS has been associated with elevated anti-inflammatory markers (e.g., IL-10) [8,134], it has been hypothesized that fatigue may not be steered directly by inflammatory status, but maybe rather by sleep disturbances which likewise result in disturbed or shifted cytokine release [134]. According to a 44% reduction in fatigue level, Zick et al. observed a 50% improvement in sleep quality [120]. In particular, the continuous recurring IL-6 is suggested to be the somnogenic factor in daytime sleepiness and fatigue, as well as IL-1 in response to foregoing infections [135,136]. However, the cause–effect relationship is not clear. Do sleep disturbances provoke a higher cytokine release which further results in fatigue and daytime sleepiness, or are sleep disturbances induced by a higher cytokine release? 

### 4.2. Future Perspectives

Further human trials are ongoing in which the effect of omega-3 FA intake in patients with cancer [137] and probiotics in patients with MS [138] on fatigue, albeit not as primary endpoint, is studied. Future trials should also expand to other probable anti-inflammatory dietary interventions. Interestingly, it appears that omega-6 FAs are not always as deleterious as believed and might instead have anti-inflammatory properties, albeit via different pathways [43,139]. Although no inflammatory markers have been explored, treatment with 3 g conjugated LA daily or placebo for 6 weeks in 33 patients with rectal cancer undergoing therapy resulted in remarkable improvements of fatigue symptoms and QoL [140]. However, supplementation of the omega-6 FA gamma-LA for 6 months in 90 patients with Sjögren’s syndrome was not effective in improving fatigue levels [141]. Possibly, it is not only a question of “what to eat”, but also “what to avoid”, as, for example, caloric restriction was able to show immune-inflammatory enhancing effects [142]. More research is needed to a) clarify specific anti-inflammatory properties and b) to verify potential positive effects on perceived disease-related fatigue. Indeed, long-term treatment durations might be considered to reach positive results [98]. Since there seems to be a direct relation between subjectively experienced fatigue and objectively measured muscle fatigue [1,11], future trials should include muscle performance as an objective endpoint to complement the subjectively perceived fatigue levels. In this context, specific blood analysis techniques might also become interesting for objectively diagnosing CFS [143].

In patients with cancer, high doses of anti-oxidative (multi) vitamins normally have to be avoided to minimize risk of dose-related side effects, as well as interferences with drug effectiveness. Interestingly, current investigations show that there are new techniques available (including nanoparticles) that make it possible to apply higher vitamin doses to treat fatigue without negatively affecting chemotherapy [144]. Nonetheless, adverse side effects are more often described in administration of high-dose single nutrients than in the context of a combined therapy or whole diet [86]. 

Regardless of disease background, fatigue is highly complex and often of unknown origin. Although IL-6 is frequently mentioned as major determinant, it is more likely that fatigue is dominated by a profound upregulated and imbalanced inflammatory network, and not dominated by only one elevated cytokine [27,145,146]. Though not via direct (anti-) inflammatory pathways, further nutrients such as coenzyme Q10 [147,148], vitamin B12 [149], and various other B-vitamins [150] are suggested to play a relevant role in chronic fatigue, too. This is especially argued by observed mitochondrial or transporter dysfunctions in CFS, which consequentially decrease energy production [151]. Furthermore, synergistic effects can be assumed, which argue for a combined treatment in terms of a whole anti-inflammatory diet rather than consumption of single anti-inflammatory nutritive agents. In this context, the highest success rates in fatigue management will probably be achieved by a multimodal lifestyle strategy consisting of various (pharmacological and nonpharmacological) approaches, including anti-inflammatory diet, exercise, and psychological support [46]. 

### 4.3. Limitations 

This is a scoping review which despite extensive literature research might have missed relevant studies. 

## 5. Conclusions

**Figure 2:**
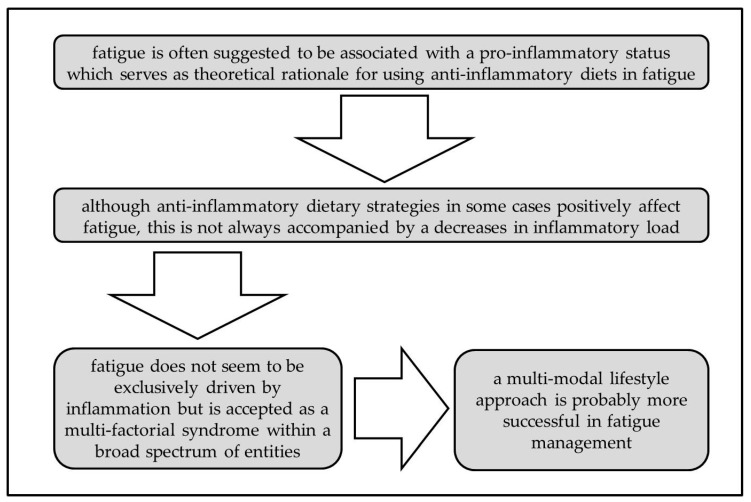
Rationale and challenges for anti-inflammatory diets in fatigue.

To the best of our knowledge, this is the first review summarizing the effects of anti-inflammatory dietary strategies on disease-related and idiopathic fatigue. Different anti-inflammatory nutritional approaches have been tested in various patient populations with fatigue, although studies are of inconsistent quality. Single nutritional approaches with, for example, high doses of vitamins were not always successful in reducing fatigue in the various patient populations. Nutritional interventions as complementary therapies are still quite new in the treatment of fatigue and data is lacking to support the hypothesis that vitamin and mineral supplementation, or any other anti-inflammatory nutrient, could robustly be effective in reducing fatigue symptoms. Furthermore, changes in inflammation and fatigue levels did seldom occur simultaneously. Therefore, although fatigue often has been suggested to be due to inflammation, a clear cause–effect relationship still has to be shown. This could explain why anti-inflammatory nutrients/dietary patterns are not always as effective in fatigue improvement as hypothesized. Moreover, a multifactorial etiology is likely, so that a multimodal approach (including nutritional intervention, physical activity, and psychological support among other possible nonpharmacological strategies) is necessary (Figure 2). 

## Abbreviations

AA	arachidonic acid
CFS	chronic fatigue syndrome
CNS	central nervous system
CRF	cancer-related fatigue
CRP	c-reactive protein
DHA	docosahexaenoic acid
EGCG	epigallocatechin gallate
EPA	eicosapentaenoic acid
ESR	erythrocyte sedimentation rate
FA	fatty acids
IBD	inflammatory bowel disease
IBS	irritable bowel syndrome
IgA/M	immungobulin A/M
IL	interleukin
IU	international units
LA	linoleic acid
LBP	LPS-binding protein
LPS	lipopolysaccharide
ME	myalgic encephalomyelitis
MS	multiple sclerosis
NAIOSs	natural anti-inflammatory and anti-oxidative substances
NF-κB	nuclear factor kappa beta
ONS	oral nutritional supplement
PUFA	poly-unsaturated fatty acid
QoL	quality of life
RA	rheumatoid arthritis
RCT	randomized controlled trial
SF-36	a widely used QoL-measurement
SLE	systemic lupus erythematosus
TNF-α	tumor necrosis factor alpha

## Appendix A

**Table A1:** Clinical trials examining anti-inflammatory dietary approaches in fatigued patients.

	Study Design	Patient Sample	Treatment	Inflammation	Fatigue	Quality of Life	Commentary	References
**Anti-inflammatory nutrients**								
Poly-unsaturated fatty acids (PUFA)	Randomized controlled trial (RCT)	*n* = 92 (♂43 ♀49) advanced non-small-cell lung cancer (NSCLC); under treatment	Intervention (*n* = 46): daily 2 cans oral nutritional supplement (ONS) with eicosapentaenoic acid (EPA) (2.2 g) vs. Control (*n* = 46): isocaloric diet duration: during first 2 cycles of chemotherapy (median 5.8 (0.3–18.4) months)	↓interleukin (IL) -6 (*p* = 0.602) ✓tumor necrosis factor (TNF)-α (*p* = 0.541) ✓c-reactive protein (CRP) (*p* = 0.07)	✓EORTC QLQ-C30 - fatigue symptoms (*p* = 0.04)	⊘EORTC QLQ-C30 - global (*p* = 0.136)	drop-out: 8 (Intervention (I): 2/ Control (C): 6) compliance: 73% EPA-group had sign. higher energy and protein intake compared to control	[39]
	phase III RCT 5-arm design	*n* = 332 (♂181 ♀151) cachectic cancer	basic treatment: daily polyphenols (300 mg), lipoic acid (300 mg), carbocysteine (2.7g), vitamin (vit) E (400 mg), vit A (30,000 IU), and vit C (500 mg) + arm 1 (*n* = 44): daily medroxyprogesterone (500 mg) or megestrol acetate (320 mg) vs. arm 2 (*n* = 25): daily 2 cans of ONS with EPA (2.2 g) vs. arm 3 (*n* =88): daily L-carnitine (4 g) vs. arm 4 (*n* = 87): daily thalidomide (200mg) vs. arm 5 (*n* = 88): combination of all duration: 4 months	⊘IL-6 (*p* = 0.94) ⊘TNF-α (*p* =0.28)	↓ Modified Fatigue Impact Scale (MFSI)-short form (SF) (*p* = 0.051)	⊘EORTC - QLQ-C30 (*p* = 0.29) ↓EQ-5D-Index (*p* = 0.002) ⊘EQ-5D-VAS (*p* = 0.79)	drop-out: 12 (arm 1: 2/ arm 2: 2/ arm 3: 3/ arm 4: 3/ arm 5: 2)	[40]
	multi-center double-blind RCT	♀97 breast cancer; survivors	omega-3 (*n* = 35): daily 6 capsules EPA/docosahexaenoic acid (DHA) (1.95 g/1.35 g) vs. omega-6 (*n* = 33): daily 6 capsules soybean oil (6 g) vs. combi (*n* = 29): daily EPA/DHA (1.65 g) and soybean oil (3 g) duration: 6 weeks	n/a	SI-CRF ✓omega-3: (*p* = 0.017) ✓omega-6: (*p* < 0.001) ✓combi: (*p* < 0.001) BFI total ✓omega-3: (*p* < 0.001) ✓omega-6: (*p* < 0.001) ✓combi: (*p* < 0.001) MFSI-SF ✓omega-3: (*p* < 0.001) ✓omega-6: (*p* < 0.001) ✓combi: (*p* < 0.001)	-	drop-out: 16 (omega-3: 5/ omega-6: 6/ combi: 5) compliance: 84.5% benefits on fatigue were more in favor of omega-6 group (*p* < 0.01)	[43]
	placebo RCT	*n* = 50 (♂8 ♀42) Systemic Lupus Erythematosus (SLE); median age 38.8 (19.8–57.7) years	Intervention (*n* = 25): daily 6 capsules EPA/DHA (2.25 g/2.25 g) vs. placebo (*n* = 25) duration: 6 months	✓ erythrocyte sedimentation rate (ESR) (*p* = 0.008) ✓IL-13 (*p* = 0.033) ✓IL-12 (*p* = 0.058)	⊘Fatigue Severity Scale (FSS) (*p* = 0.350) ⊘SF-36 - energy/ fatigue (*p* = 0.092)	⊘SF-36 - emotional wellbeing (*p* = 0.070)	drop-out: 18 (I: 7/placebo (P): 11) compliance: I: 83%/ P: 64%	[49]
	double-blind, open-label RCT	*n* = 27 (♂4 ♀23) Multiple Sclerosis (MS); relapsing- remitting	basic treatment: daily vit E (400 international units (IU)), multi-vitamins, calcium (500 mg) + Intervention (*n* =14): low-fat diet (15energy%) and daily 6 capsules EPA/DHA (1.98 g/1.32 g) vs. Control (*n* = 13): normal-fat AHA Step I diet (≤ 30energy%) and placebo capsules (olive oil) duration: 1 year	⊘IL-4 (n/a) ⊘IL-8 (n/a) ⊘IL-12 (n/a) ⊘IL-1ß (n/a) ⊘interferon (IFN)-γ (n/a) ⊘TNF-α (n/a)	⊘MFIS (*p* = 0.059)	⊘SF-36 (n/a) ⊘MHI (n/a)	drop-out: 8 (I: 2/ C: 6) compliance: I: 69.2%/ C: 66.7% benefits on fatigue were more in favor of placebo-group and were related to increased DHA-levels	[50]
	multi-center, double-blind, placebo RCT	*n* = 91 (♂32 ♀59) MS; relapsing- remitting	basic treatment: 3x/wk Rebif (44 µg) + Intervention (*n* = 46): daily 5 capsules EPA/DHA (1.35 g/0.85 g) vs. placebo (*n* = 45): corn oil (5 g) duration: 6 months	-	⊘FSS (*p* =0.97)	⊘SF-36 (*p* = 0.53)	drop-out: 11 (I: 3/ P: 8)	[51]
Anti-oxidative vitamins	double-blind, placebo RCT	*n* = 101 (♂25 ♀76) MS; relapsing- remitting	Intervention (*n* = 51): daily vit A (first 6 months 25,000 IU, then 10,000 IU) vs. placebo (*n* = 50) duration: 12 months	-	✓MFSI (*p* = 0.004)	-	drop-out: 8 (I: 4/ P: 4)	[55]
Vitamin D	double-blind, placebo RCT	*n* = 45 (♂25 ♀76) juvenile SLE	Intervention (*n* = 22): 1x/wk oral cholecalciferol (50,000 IU) vs. placebo (*n* = 23) duration: 24 weeks	ESR? CRP?	✓kids-FSS (*p* = 0.012)	-	drop-out: 5 (I: 2/ P: 3) compliance: I: 85%/ P: 75%	[64]
Polyphenols	phase I uncontrolled study	*n* = 12 (♂11 ♀1) advanced/ metastatic kidney cancer; under treatment; median age 63.3 (48–83) years	Intervention: 2x/day isoquercentin (450 mg), vit C (111.6 mg), vit B3 (9 mg) vs. Control: 2x/day isoquercentin (225 mg), vit C (55.8 mg), vit B3 (4.5 mg) duration: during 2 or 4 sunitinib cycles (median 81 (75.5–86.5) days)	-	✓ Functional Assessment of Chronic Illness Therapy Fatigue (FACIT-F) (*p* = 0.002)	⊘ Functional Assessment of Cancer Therapy (FACT)-General (*p* = 0.069)	safety trial	[72]
Protein/Amino acids	phase III RCT 5-arm Design	*n* = 332 (♂181 ♀151) cachectic cancer	basic treatment: daily polyphenols (300 mg), lipoic acid (300 mg), carbocysteine (2.7 g), vit E (400 mg), vit A (30,000 IU), and vit C (500 mg) plus arm 1 (*n* = 44): daily medroxyprogesterone (500 mg) or megestrol acetate (320 mg) vs. arm 2 (*n* = 25): daily 2 cans of ONS with EPA (2.2 g) vs. arm 3 (*n* = 88): daily L-carnitine (4 g) vs. arm 4 (*n* = 87): daily thalidomide (200mg) vs. arm 5 (*n* = 88): combination of all duration: 4 months	⊘IL-6 (*p* = 0.663) ⊘TNF-α (*p* = 0.24)	⊘MFSI-SF (*p* = 0.801)	⊘EORTC - QLQ-C30 (*p* = 0.832) ⊘EQ-5D-Index (*p* = 0.151) ⊘EQ-5D-VAS (*p* = 0.593)	drop-out: 12 (arm 1: 2/ arm 2: 2/ arm 3: 3/ arm 4: 3/ arm 5: 2)	[40]
	exploratory open-label RCT	*n* = 90 (♂21 ♀69) chronic fatigue syndrome (CFS)	ALC (*n* = 30): daily acetyl-L-carnitine (2 g) vs. PLC (*n* = 30): daily propionyl-L-carnitine (2 g) vs. ALC+PLC (*n* = 30): daily acetyl-L-carnitine (2 g) + propionyl-L-carnitine (2 g) duration: 24 weeks	-	MFI-20 ⊘ALC: (*p* = 0.218) ✓PLC: (*p* = 0.004) ✓ALC+PLC: (*p* < 0.001)	-	drop-out: 19 (ALC: 9/ PLC: 4/ ALC+PLC: 6)	[84]
**Anti-inflammatory foods**								
Probiotics	double-blind, placebo RCT	*n* = 28 (♂13 ♀15) irritable bowel syndrome (IBS); mean age 50 ± 8.6 years	high-dose (HD) (*n* = 8): daily 2 capsules synbiotics (*Lactobacillus*/*Bifidobacterium*) vs. low-dose (LD) (*n* = 10): daily 1 capsule synbiotics + 1 placebo vs. placebo (*n* = 10): daily 2 capsules duration: 8 weeks	-	⊘FSS (*p* = 0.115) ✓MFI-20 (*p* = 0.037) ✓Fatigue-VAS (*p* = 0.013)	-	drop-out: 2 (HD: 2/ LD: 0/ P: 0); dose-dependent relationship	[105]
	uncontrolled, open pilot study	*n* = 15 (♂5 ♀10) CFS; median age 43 (30–56) years	Intervention (*n* = 15): 2x/day probiotic yoghurt (*L. paracasei*, *L. acidophilus*, *B. lactis*) duration: 8 weeks	-	⊘Fatigue-VAS (n/a)	⊘SF-12 - health (n/a)	compliance has been checked via faecal samples	[106]
	double-blind, placebo RCT	*n* = 60 (♂35 ♀25) colorectal cancer; completed treatment; mean age 56.2 ± 8.9 years	Intervention (*n* = 32): 2x/day Lacidofil-pills (*L. rhamnosus*, *L. acidophilus*) vs. placebo (*n* = 28) duration: 12 weeks	-	⊘FACT - Fatigue (*p* = 0.09)	⊘FACT - General (*p* = 0.14) ✓FACT - cancer- related (*p* = 0.004) ✓FWB (*p* = 0.04)	drop-out: 6 (I:5/ P:1)	[107]
	double-blind, cross-over RCT	*n* = 14 (♂14 ♀1) primary sclerosing cholangitis (PCS) and IBD; median age 45 (28-70) years	Intervention: 2x/day probiotic sachet (*L. acidophilus*, *L. casei*, *L. salivarius*, *L. lactis*, *B. bifidum*, *B. lactis*) vs. placebo duration: 12 weeks	-	⊘Fatigue-VAS (n/a)	-	drop-out: 2 cross-over after 3 months	[108]
	double-blind RCT	*n* = 63 (♂40 ♀23) spondyloarthritis, active disease; median age 43.3 (19–76) years	Intervention (*n* = 32): 2x/day oral probiotic powder (*S. salivarius*, *B. lactis*, *L. acidophilus*) vs. placebo (*n* = 31) duration: 12 weeks	⊘CRP (n/a)	⊘Multi-dimensional Assessment of Fatigue (MAF) (n/a)	⊘ASQoL (n/a)	drop-out: 0; compliance was checked by weighing powder	[109]
Root plants	single-blind RCT	*n* = 66 (♂56 ♀10) non-alcoholic fatty liver disease (NAFLD); mean age 49.9 ± 12.2 years	basic treatment: daily 1 capsule *Silybum marianum* (450 mg), 30min. aerobic exercise + Intervention (*n* = 35): 3x/day capsule ginsenosides (3 g) vs. placebo (*n* = 31) duration: 3 weeks	✓TNF-α (*p* = 0.031) ⊘IL-6 (*p* = 0.227)	⊘FSS (*p* = 0.221)	-	drop-out: 14 (I: 5/ P: 9) patients with BMI ≥ 25 showed pronounced benefit regarding TNF-α and fatigue	[114]
	double-blind RCT	*n* = 90 (♂21 ♀69) CFS; median age 39.5 (20–60) years	HD (*n* = 30): 2x/day *P. ginseng* (2 g) vs. LD (*n* = 30): 2x/day *P. ginseng* (1 g) vs. placebo (*n* = 30)		⊘NRS total (*p* = 0.068) NRS mental ✓LD: (*p* = 0.033) ✓HD: (*p* = 0.002) Fatigue-VAS ⊘LD: (*p* = 0.449) ✓HD: (*p* = 0.037)		drop-out: 2 (HD: 1/ LD: 1/ P: 0) dose-dependent amelioration	[115]
	double-blind RCT	*n* = 51 (♂19 ♀32) cancer; under treatment; mean age 58 ± 12 years	Intervention (*n* = 24): 4x/day ginger extract (1.2 g; incl. gingerols 60mg) vs. placebo (*n* = 27) duration: during first 3 cycles of chemotherapy		✓FACIT-Fatige (*p* = 0.013)	✓FACT - General (*p* = 0.040)	drop-out: 17 (I: 9/ P: 8)	[117]
**Anti-inflammatory dietatry patterns**								
Fatigue reduction diet	pilot RCT	♀30 breast cancer survivors; mean age 62.4 ± 9.7 years	Intervention (*n* = 15): fatigue reduction diet vs. Control (*n* = 15): general health curriculum duration: 3 months	-	✓ Brief Fatigue Inventory (BFI) (*p* = 0.01)	-	compliance/ efficacy checked by dietary records and serum levels; control group sign. younger and leaner	[120]
Leaky gut diet	uncontrolled study	*n* = 41 (♂7 ♀34) CFS; mean age 37.9 ± 11.1 years	Intervention: leaky gut diet + NAIOs duration: 10–12 months	✓LPS-immuno-globulin (Ig)M (*p* = 0.0002) ✓LPS-IgA (*p* = 0.0009)	✓FF scale (*p* = 0.0002)	-	63.5% responders; more benefit with shorter illness duration, younger age	[98]

*n* number ♂ male ♀ female ✓ improvement ↓ deterioration ⊘ no effect n/a not available AHA American Heart Association ALC acetyl-L-carnitine ASQoL Ankylosing Spondylitis Quality of Life Questionnaire BFI Brief Fatigue Inventory BMI body mass index C: control CFS chronic fatigue syndrome CRP c-reactive protein DHA docosahexaenoic acid en% energy percent EORTC-QLQ European Organization for the Research and Treatment of Cancer - Quality of Life Questionnaires EPA eicosapentaenoic acid EQ-5D European Quality of Life 5 Dimensions ESR erythrocyte sedimentation rate FA fatty acid FACIT Functional Assessment of Chronic Illness Therapy FACT Functional Assessment of Cancer Therapy FF scale Fibromyalgia and Chronic Fatigue Syndrome Rating Scale FSS Fatigue Severity Scale FWB Functional Well-Being HD high-dose I: intervention IBD inflammatory bowel disease IBS irritable bowel syndrome IL interleukin IFN-γ interferon gamma IU international units LD low-dose LPS lipopolysaccharide LPS-IgM/A immunoglobulin M/A responses to lipopolysaccharide MAF Multidimensional Assessment of Fatigue MFI-20 Multidimensional Fatigue Inventory MFIS Modified Fatigue Impact Scale MFSI-SF Multidimensional Fatigue Symptom Inventory–Short Form MHI Mental Health Inventory MS Multiple Sclerosis NAFLD non-alcoholic fatty liver disease NAIOs natural anti-inflammatory and anti-oxidative substances NRS self-rating numeric scale for fatigue severity NSCLC non-small cell lung cancer ONS oral nutritional supplement P: placebo PCS primary sclerosing cholangitis PG1/2 Panax ginseng 1g/2g PLC propionyl-L-carnitine PUFA poly-unsaturated fatty acid RCT randomized controlled trial SF-36 Health Survey SI-CRF Symptom Inventory cancer-related fatigue SLE Systemic Lupus Erythematosus TNF-α tumor necrosis factor alpha VAS Visual Analogue Scale vit vitamin wk week.
